# A Semi-Three-Dimensional Bioprinted Neurocardiac System for Tissue Engineering of a Cardiac Autonomic Nervous System Model

**DOI:** 10.3390/bioengineering10070834

**Published:** 2023-07-14

**Authors:** Ivana Hernandez, Salma P. Ramirez, Wendy V. Salazar, Sarahi Mendivil, Andrea Guevara, Akshay Patel, Carla D. Loyola, Zayra N. Dorado, Binata Joddar

**Affiliations:** 1 Inspired Materials and Stem-Cell Based Tissue Engineering Laboratory (IMSTEL), The University of Texas at El Paso, El Paso, TX 79968, USA; 2 Department of Metallurgical, Materials and Biomedical Engineering, M201 Engineering, The University of Texas at El Paso, 500 W. University Avenue, El Paso, TX 79968, USA; 3 Department of Chemical Engineering, University of California, Santa Barbara, CA 93106, USA; 4 Border Biomedical Research Center, The University of Texas at El Paso, 500 W. University Avenue, El Paso, TX 79968, USA

**Keywords:** 3D bioprinting, neurocardiac junctions, 3D tissue model, cardiomyocytes, neuroblastoma cells

## Abstract

In this study, we designed a tissue-engineered neurocardiac model to help us examine the role of neuronal regulation and confirm the importance of neural innervation techniques for the regeneration of cardiac tissue. A three-dimensional (3D) bioprinted neurocardiac scaffold composed of a mixture of gelatin–alginate and alginate–genipin–fibrin hydrogels was developed with a 2:1 ratio of AC16 cardiomyocytes (CMs) and retinoic acid-differentiated SH-SY5Y neuronal cells (NCs) respectively. A unique semi-3D bioprinting approach was adopted, where the CMs were mixed in the cardiac bioink and printed using an anisotropic accordion design to mimic the physiological tissue architecture in vivo. The voids in this 3D structure were methodically filled in using a NC–gel mixture and crosslinked. Confocal fluorescent imaging using microtubule-associated protein 2 (MAP-2) and anticholine acetyltransferase (CHAT) antibodies for labeling the NCs and the MyoD1 antibody for the CMs revealed functional coupling between the two cell types in the final crosslinked structure. These data confirmed the development of a relevant neurocardiac model that could be used to study neurocardiac modulation under physiological and pathological conditions.

## 1. Introduction

The cardiac autonomic nervous system (CANS) controls hemodynamic changes via regulation of the heart rate, among other important functions. This is mostly because the sympathetic and parasympathetic impulses between the heart and the peripheral and central nervous systems are carefully balanced, regulating the autonomic control of the heart through multiple levels of feedback loops [1,2]. More specifically, the sympathetic ganglia combine central and peripheral inputs to send signals to the heart through motor neurons that target cardiomyocytes. The manner in which neural information is transmitted to the corresponding cardiac targets is determined by this communication. The relationship between these types of cells, and cardiomyocytes and neurons has not yet been comprehensively studied [3] in the context of ischemic heart disease or chronic coronary syndrome, which are a few of the most prevalent causes of heart failure. This condition lowers blood oxygen levels and weakens the myocardial tissue and contractile forces generated [4]. There are currently two common approaches that are adopted to counteract the effects of this disease, one being the use of antianginal drugs. The other technique entails surgical interventions such as coronary angiography, followed by either percutaneous coronary intervention or coronary artery bypass grafting [5]. Although not prevalent, engineering cardiac tissue with neuronal innervation also seems to be a potential therapeutic strategy towards the treatment of ischemic heart disease. 

Thus, in this study, our goal was to co-culture cardiomyocytes and neuronal cells in a 3D model that could be successfully extrapolated towards the biofabrication of a CANS model using bioprinting [6,7]. It is hypothesized that such a CANS model tissue-engineered in vitro could eventually strengthen our understanding and pave the way for the development of a regenerative medicine-based strategy for the treatment of ischemic heart disease in clinical settings. In this present work, an original approach using a semi-three-dimensional (3D) bioprinting strategy was adopted to fabricate a neurocardiac scaffold made of two different bioinks. The cardiac bioink was composed of gelatin–alginate and contained AC16 cardiomyocytes (CMs), and the neuronal bioink was composed of alginate–genipin–fibrin and retinoic acid-differentiated SH-SY5Y neuronal cells (NCs). AC16 CMs have emerged as a promising cell line for the development of cardiac tissue constructs via extrusion-based 3D bioprinting, enduring the harsh conditions associated with this method, such as shear stress, without losing their functionality [8,9]. Similarly, SH-SY5Y NCs is a neuroblastoma cell line and has been widely used in neuroscience and toxicology research. These cells have also demonstrated compatibility with the components of the bioink utilized in this study, as well as their capability to remain viable after printing and crosslinking processes [10]. 

The neurocardiac scaffold was developed using a mixture with a 2:1 ratio of both cell types (cardiomyocytes: neurons) to improve the heterocellular interactions between both cell lines, as reported in previously published studies from another group [7]. Such studies have investigated neurocardiac interactions using an aspect ratio where the cardiomyocytes outnumber neuronal cells, demonstrating a functional and viable co-culture [7,11,12]. Although the distribution and numbers present in cardiac tissue have not been extensively studied, intrinsic cardiac neurons are distributed in a similar pattern and form a limited number of clusters within the cardiac tissue [13]. 

By incorporating multiple arrowheads into the design, the scaffold design was intended to replicate the geometry and morphology of myocardial tissue [6], and adding an even layer with neuronal cells potentially mimicked the organization of a CANS model [13]. It was expected that this design would promote effective networking between neurons and cardiomyocytes in comparison with a 2D platform [7]. Cell growth, function, maintenance, and heterocellular coupling would have steadily risen in all regions of the structure as a result of the circulation of the mixed growth media from the interior to the exterior of the scaffold design [8], allowing effective communication between the two cell types and leading to the formation of a tissue-engineered CANS model. 

## 2. Materials and Methods

### 2.1. Materials

For making the cardiac bioink, the following materials and supplies were used: medium-viscosity alginate (MVG) (Sigma Aldrich, Victoria, BC, Canada), gelatin (Sigma-Aldrich, Victoria, BC, Canada), and 3 mL syringe-printing cartridges with a 20G tapered conical nozzle (CELLINK, Blacksburg, VA, USA). For the cardiac bioink crosslinking solution, 1X PBS (Fisher Bioreagents) and calcium chloride dihydrate (Fisher Chemical, Schwerte, Germany) were used. To create the ionic crosslinking solution, calcium chloride (CaCl_2_) was added into 1X PBS with a final concentration of 100 mM. 

For making the neuronal bioink, low-viscosity alginate (LVG) was used with 1X Tris-buffered saline (Component 1), 25 mg/mL genipin (Component 2), and 50 mg/mL fibrinogen (Component 3). The crosslinker’s components also included 25 mg/mL chitosan with approximately 560 mg/mL β-glycerophosphate (Component A) and 1000 U/mL thrombin with 20 mg/mL calcium chloride (Component B). Components 1–3 and A–B were provided by Axolotl Sciences (Victoria, BC, Canada) and were developed previously by the Willerth Lab at the University of Victoria [14,15]. The following equipment and reagents were used for all cellular experiments: plastic petri dishes (ThermoFisher Scientific, Waltham, MA, USA); 1X PBS (Cytiva HyClone™, Logan, UT, USA); serological pipettes with sizes of 1 mL, 5 mL, and 10 mL; and 6-well plates (ThermoFisher Scientific, Waltham, MA, USA). The crosslinker’s purpose for both bioinks was to sequester the structure together so that the shape would hold when exposed to non-uniform stress. 

Please refer to Supplementary Section S2.1 for further details on the materials used for making the bioinks, for the cell culture and passaging, as well as for immunostaining. 

### 2.2. Preparation of the Cardiac Bioink

The cardiac bioink consisted of medium-viscosity alginate (MVG) mixed with gelatin to make it extrudable under moderate amounts of shear pressure. The bioink contained 10% (*w*/*v*) gelatin dissolved in PBS (pH 7.4) with light heat and constant mixing with a magnetic stirrer under sterile conditions. Another solution of 14% (*w*/*v*) MVG sodium alginate was made with or without the cell suspension in combined growth media in a 5 mL conical tube. Both solutions were combined into a separate 5 mL conical tube so that the absolute concentration of the bioink was 5% (*w*/*v*) gelatin and 7% (*w*/*v*) MVG alginate [8]. The mixture was allowed to dissolve overnight at room temperature and then centrifuged at 1200 rpm for 3 min to remove air bubbles. All gels were UV-sterilized for 15 min before loading into printing cartridges or mixing with the cells. For cell-based studies, this cardiac bioink was mixed with the cells and loaded into a 3 mL syringe-printing cartridge with a 20G tapered conical nozzle. Acellular gels were loaded without mixing cells for material characterization studies. To create the ionic crosslinking solution, calcium chloride (CaCl_2_) was added into 1X PBS with a final concentration of 100 mM. 

### 2.3. Preparation of the Neuronal Bioink 

The neuronal bioink was prepared according to previously published works [9,10]. All reagents were previously prepared, sterilized, and provided by Axolotl Biosciences (Victoria, BC, Canada). Upon being received, the components (see Section S2.1) were stored at −20 °C. Bioink Component 1 and Crosslinker Components A and B were thawed at 4 °C, while bioink Components 2 and 3 were thawed at room temperature and at 37 °C, respectively. For the acellular experiments, bioink Component 2 at a concentration of 0.3 mg/mL was pipetted into Component 1 (0.5% *v*/*v*). After mixing these reagents, Component 3 (20 mg/mL) was slowly pipetted into the mixture and was once again mixed. For bioprinting, the cells were mixed with Component 3 and then the cell mixture was added to the mixture of Components 1 and 2. For the crosslinker solution, Component B (thrombin, 1.7 U/mL; calcium chloride, 19.16 mg/L) was slowly added with a pipette to Component A (chitosan, 19 mg/mL; β-glycerophosphate, 6.6 mg/mL). Please refer to Supplementary Section S2.1 for the details of all components of the neuronal bioink. SH5YSY cells have been co-formulated and deposited as biosensors with other compounds in existing studies so we hypothesized these cells to be viable in the bioink during the bioprinting process in this study [16]. 

### 2.4. Semi-3D Bioprinting 

SolidWorks software (v2022 SP02) was used to design a 3D accordion-like arrowhead scaffold with dimensions of 20 mm (L) × 20 mm (W) × 0.05 mm (H), a strut thickness of 0.5–1 mm, and spaces of 1 × 2.12 mm, which was bioinspired by the honeycomb-like lattice framework of an in-vivo heart tissue [8,17]. The cardiac bioink was directly extruded into this design onto a 100 × 15 mm petri dish utilizing a BIOX (CELLINK, Blacksburg, VA, USA) 3D bioprinter. The optimal bioprinting settings [18] and parameters can be seen in Table 1. 

The complexity of the scaffold’s design was taken into consideration when choosing the printing speed, the nozzle’s diameter was selected based on the thickness of the design, and the printing pressure and temperature were set on the basis of how easily the bioink was extruded, thus preventing shear stress and increasing cell viability [19]. For bioprinting with cells, the cardiac bioink was mechanically mixed with 2 × 10^6^ cells/mL in a 5 mL conical tube containing 2 mL of bioink and loaded into a sterile 3 mL syringe using a female Luer lock connector for printing with the parameters found in Table 1. The neuronal bioink was made by mixing 1 × 10^6^ cells/mL in a 5 mL conical tube containing 3 mL of the bioink. The neuronal bioink laden with cells was deposited on top of the cardiac scaffold using a multichannel pipette and allowed to settle and penetrate the bottom cardiac layer. The bilayered composite structure was then crosslinked using a crosslinking mixture for both bioinks mixed in a ratio of 2:1 (cardiac:neuronal). For fluorescent imaging, cells were pre-stained with the PKH67 Green Fluorescent Cell Linker Mini Kit for differentiated SH-SY5Y cells and the PKH26 Red Fluorescent Cell Linker Mini Kit for AC16 cardiomyocytes by following the vendor’s recommendations. The 3D bioprinted neurocardiac structures were kept immersed in 1 mL of the combined media (2:1 cardiac:neuronal ratio) during all culture times. 

Following the 3D printing of the cardiomyocyte-specific structure, the neuronal bioink was micropipetted on top of the cardiac scaffold, dispersed evenly throughout the printed surface, and left to sit for a total of 2 min. After fabricating the co-culture layout, the semi-3D printed structure was crosslinked first with the neuronal crosslinker and removed after 1 min, then sufficient cardiac crosslinker (100 mM CaCl_2_) was added with a pipette to cover the whole structure and placed on a Belly Dancer shaker (IBI SCIENTIFIC, Dubuque, IA, USA) at 10 rpm. Once the crosslinker had been removed after 5 min, the composite crosslinked structure was washed with 1X PBS three times and placed into a 6-well plate for further experimentation. 

### 2.5. Material Characterization

For details on the swelling analysis (Supplementary Section S2.5.1), ATR-FTIR (Supplementary Section S2.5.2), and SEM (Supplementary Section S2.5.3) please refer to Supplementary Section S2.5.

### 2.6. Cell Culture and Passaging

For details on the cell culture and passaging, please refer to Supplementary Section S2.6.

### 2.7. Fluorescent Imaging

#### 2.7.1. Heterocellular Coupling

The total number of heterocellular couplings between the PKH26-stained (red) AC16 cardiomyocytes and the PKH67-stained (green) SHSY-5Y differentiated neuronal cells was assessed in order to analyze the interactions between both cell lines that were placed in the semi-3D bioprinted structures via confocal microscopy imaging. The cells were first fixed by immersing the structures in a 4% paraformaldehyde (PFA) solution for 15 min at room temperature. The structures were then rinsed thrice with 1X PBS and placed on a Belly Dancer Shaker at 10 rpm for 5 min. The samples were counterstained using mounting media with DAPI and placed on glass slides prior to imaging. The average percentage of heterocellular cell coupling was calculated by using Equation (1), where the number of overlapping regions from the interactions between the CMs (red) and NCs (green) was counted.

(1) $$\%\;\mathrm{Coupling}=\frac{\#\;\mathrm{of}\;\mathrm{overlapping}\;\mathrm{CMs}\;\left(\mathrm{red}\right)\;\mathrm{and}\;\mathrm{NCs}\;(\mathrm{green})}{\mathrm{total}\;\#\;\mathrm{of}\;\mathrm{cells}\;\mathrm{in}\;\mathrm{image}}\times 100$$

#### 2.7.2. Immunostaining

Immunostaining studies were performed to detect the expression of MyoD1 in the AC16 cardiomyocytes, and MAP-2 and CHAT in the differentiated SH-SY5Y neurons [20,21]. The AC16 cardiomyocytes in the 3D-printed structures were washed with 1X PBS three times and fixed with a 4% paraformaldehyde (PFA) solution for 15 min at room temperature. The structures were rinsed 3 times, immersed in 1X PBS, and placed on a Belly Dancer Shaker at 10 rpm for 5 min. Cells were blocked with a blocking solution overnight at 4 °C. After removal of the blocking solution, the structures were rinsed thrice using 1X PBS and placed on a Belly Dancer Shaker for 5 min. The primary antibodies were then added to the samples and incubated overnight at 4 °C. The samples were then washed three times with 1X PBS and then incubated for 1 h at room temperature with the secondary antibodies as outlined earlier in Section 2.1. The secondary antibody solution was then removed, and the samples were washed again and mounted on glass slides, and images were acquired using a confocal fluorescent microscope.

## 3. Results

**Figure 1:**
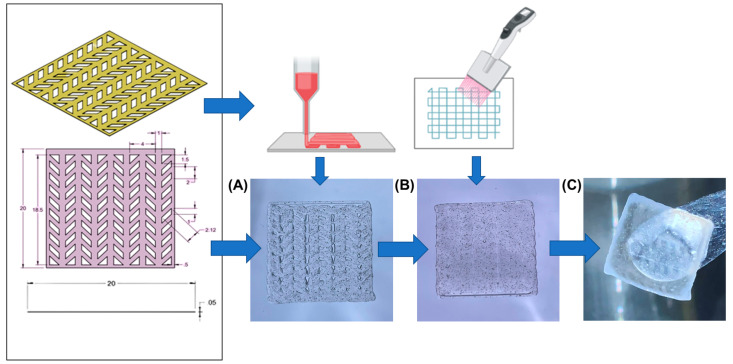
Semi-3D printed neurocardiac structure. The cardiac bioink was 3D bioprinted (based on the STL design shown) and placed on bottom (**A**) while the neuronal bioink was pipetted and layered on the top (**B**). The composite 3D structure after crosslinking can be observed in (**C**).

In this study, our goal was to create a 3D biological in vitro model representing the neurocardiac junctions found in the human heart. To achieve these, cardiomyocytes and neurons were co-cultured on a bilayered hydrogel structure assembled using a novel semi-3D printing approach consisting of two different bioinks containing cardiomyocytes and neurons, referred to as the cardiac and the neuronal bioink, respectively. The cardiac bioink was laden with cardiomyocytes mixed in a hydrogel mixture consisting of gelatin and alginate, and was crosslinkable by the addition of divalent calcium ions. The neuronal bioink was laden with differentiated SH-SY5Y neurons mixed alginate, genipin, and fibrin; the hydrogel was crosslinked using chitosan, β-glycerophosphate, thrombin, and calcium chloride. As shown in Figure 1, this work led to the development of a semi-3D printed cardiac–neuronal scaffold for tissue engineering applications. Briefly, the cardiac scaffold was 3D-printed and deposited using the cardiac bioink and based on the STL design depicted in Figure 1. Next, the neuronal bioink was pipetted and layered on top of the cardiac scaffold and allowed to confirm to the shape of this basal layer and fill in all the gaps. This step took a total of 1–2 min. Finally, the 3D composite bilayered structure was crosslinked using a mixture of crosslinking agents for the cardiac and the neuronal bioinks mixed at a ratio of 1:1. The crosslinking step took an additional 2 min, and the endpoint of this step was determined by visual confirmation of the scaffold turning blue (confirmed by genipin crosslinking [22]), indicating the formation of the composite crosslinked cardiac–neuronal scaffold. The crosslinker’s purpose was to sequester the structure together so that the shape would hold when exposed to non-uniform stress.

**Figure 2:**
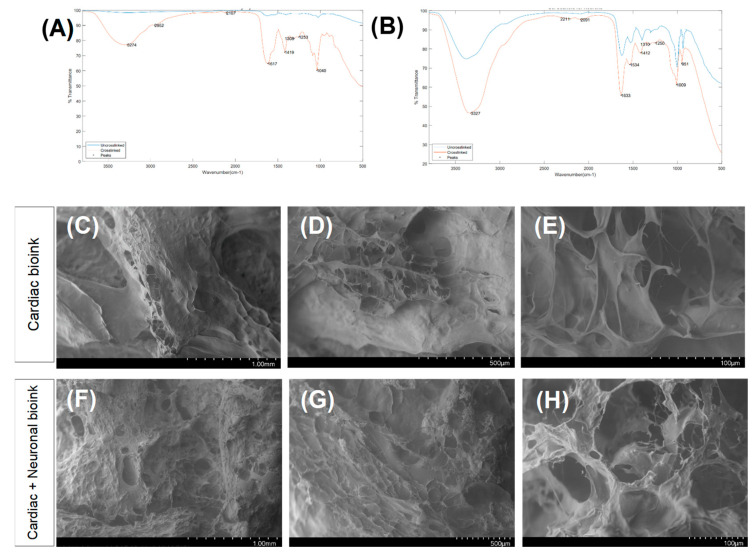
Results of the FTIR analysis for scaffolds printed using the bioink for cardiac cells (**A**) and the bioink for the neurons (**B**). A comparison between the non-crosslinked and crosslinked scaffolds, depicted in blue and red, respectively. SEM images of the bioprinted cardiac structure (**C**–**E**) and the neurocardiac scaffold (**F**–**H**).

Fourier transform infrared (FTIR) spectroscopy not only confirmed each of the hydrogels’ chemistry and crosslinking (Figure 2A,B), but it also identified the chemical compatibility between the two hydrogels. The most eminent peaks that were identified included a broad and stretched peak at ~3300 cm^−1^ that confirmed C-H and O-H stretching [23]. Another peak was evident at ~1020 cm^−1^ and corresponded to C-O-C stretching [24], whereas the relatively small peak at ~1315 cm^−1^ implied the presence of C-O stretching that is typical of alginate’s chemistry [25]. The peak at ~1620 cm^−1^ corresponded to the C=N Schiff’s base and was typical of gelatin [26]; this was detected most accurately in the crosslinked cardiomyocyte structure. The peak at ~1500 cm^−1^ pointed towards the presence of aromatic ring-related bonds and was found in the neuronal bioink, possibly due to genipin [27]. All these peaks confirmed successful crosslinking and were distinct when compared between the crosslinked and non-crosslinked structures. 

As shown in the SEM images in Figure 2C–E, the cardiac scaffolds revealed design’s specificity with the .stl file that was used to guide the accordion design of this scaffold. The pores and struts present in the cardiac scaffold were greater compared with the composite neurocardiac scaffold depicted in Figure 2F–H. In the composite neurocardiac scaffold, the voids present in the cardiac counterpart appeared to be filled in, as confirmed by the smaller pores distributed throughout the imaged sample’s surface. 

**Figure 3:**
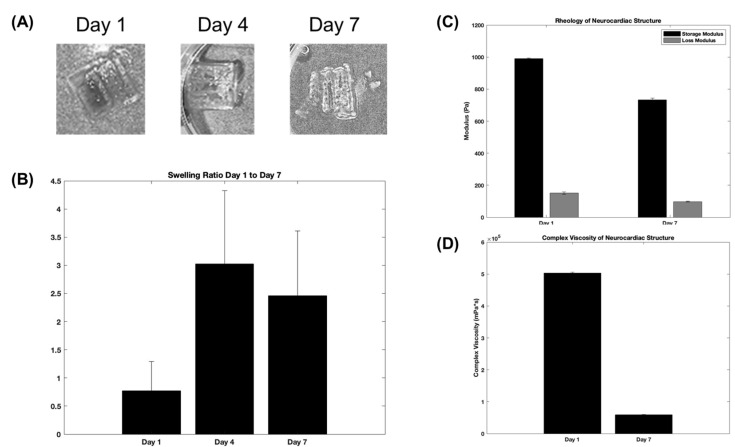
Swelling and rheological analyses. The figures show representative structures at varying time points (**A**), which are included in the graph below (**B**). (**C**) The storage and loss moduli, and (**D**) the complex viscosity plotted for the 3D bioprinted and crosslinked neurocardiac scaffold at varying time points.

Figure 3 depicts results obtained from the morphological and mechanical characterization studies of the composite and crosslinked neurocardiac scaffolds using swelling and rheological analyses, respectively. Figure 3A shows the swollen scaffolds at varying time points that are graphically represented in Figure 3B below. Both the figures and the graphs confirmed the retention of the structural fidelity of the scaffolds during the entire study period; however, the maximum swelling occurred after 4 days, and the trends seemed to suggest a sustained degradation of the structure when studied after 7 days. Figure 3C shows the storage and loss moduli, and Figure 3D shows the complex viscosity of the 3D bioprinted and crosslinked neurocardiac scaffold at varying time points. The storage modulus is a measure of how much energy a sample needs to undergo distortion, while the loss modulus accounts for the energy lost between the loading and unloading of the cyclic strain. Complex viscosity indicates the overall resistance to flow or deformation of the material, with higher values representing more viscous or less flowable materials. It is also frequency-dependent and was determined by subjecting the scaffolds to oscillatory shear stress. As shown by our results in Figure 3C,D, the reduction in the storage and loss moduli as well as the complex viscosity when assayed after 7 days confirmed the sustained biodegradation of the composite crosslinked scaffolds. However, the storage modulus was significantly higher in value compared with the loss modulus at all time points studied, confirming the presence of a viscoelastic structure that had more of the solid content compared with its liquid content. 

**Figure 4:**
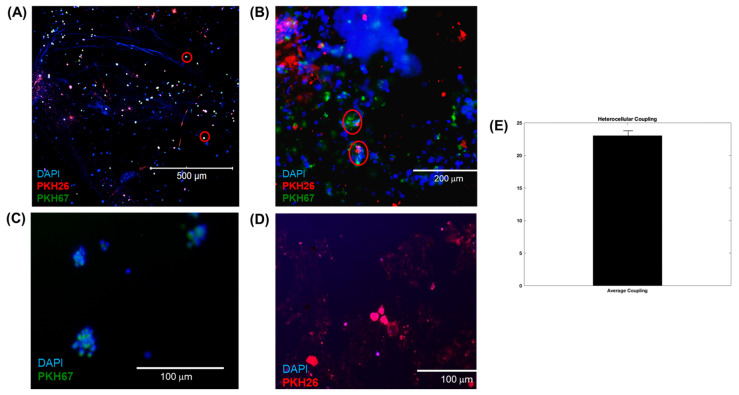
(**A**,**B**) Three-dimensional neurocardiac cultures showing an image of neuronal cells stained green (PKH67), cardiomyocytes stained red (PKH26), and nuclei stained blue (DAPI). Regions with red circles show the neurocardiac heterocellular coupling between both cell types studied. (**B**) Another representative image from a 3D neurocardiac culture at a higher magnification. (**C**,**D**) Indicative images from the 2D cultures of (**C**) neuronal and (**D**) cardiac cells. (**E**) Quantification of the heterocellular coupling shown in (**A**). Data in (**E**) are plotted as the percentage of coupling reported as the mean ± SD of all images analyzed.

Figure 4A,B shows characteristic images captured from 3D neurocardiac cultures, with the neuronal cells stained green (PKH67), the cardiomyocytes in red (PKH26), and the nuclei in blue (DAPI). Regions with an overlap of both colors (red circles) confirmed the heterocellular neurocardiac coupling between both cell types studied. Figure 4C,D shows representative images from the 2D cultures of the neuronal and cardiac cells. These results confirmed the neurocardiac coupling relationship in the 3D crosslinked scaffolds in comparison with the 2D cultures. Moreover, the cells retained their viability in the accordion-designed scaffold, which may have also helped retain the culture’s viability. Figure 4E shows the quantification of the heterocellular coupling shown in Figure 4A,B. The extent of heterocellular coupling between neuronal and cardiac cell cultures was quantified and depicted in Figure 4E. Our results depict similar trends to those reported by earlier published studies by others and by our own group, and the extent of heterocellular coupling as well as cellular viability was enhanced in the 3D scaffolds in comparison with the 2D cultures (~5%, data not included) [7,8,9,17,28,29,30]. 

**Figure 5:**
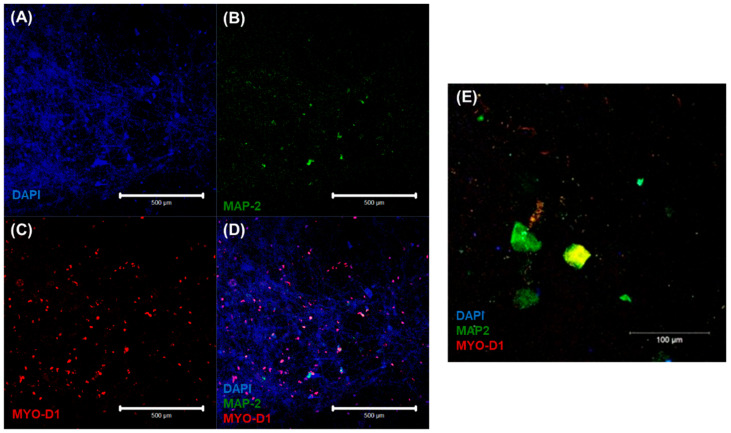
Immunostaining results confirmed via confocal microscopic imaging of 3D neurocardiac cultures (**A**–**D**) showing the AC16 cardiomyocytes (MYO D1 + Alexa Fluor 647, red) (**C**) and SH-SY5Y neurons (MAP-2 + Alexa Fluor 488, green) (**B**). All cell nuclei were stained using DAPI (blue) (**A**). The merged result of both cell types (**D**,**E**).

The characteristic cardiac and neuronal cell phenotypes and morphologies that were involved in the heterocellular cell coupling were confirmed by confocal microscopic imaging via immunostaining. Results from the 3D neurocardiac cultures are shown in Figure 5A–D, showing the AC16 cardiomyocytes (red) in Figure 5C, and the SH-SY5Y neurons (green) in Figure 5B. All cell nuclei were stained using DAPI (blue), as depicted in Figure 5A, and the merged result showing both cell types is depicted in Figure 5D,E. Cardiomyocytes were probed by using their expression of the MyoD1 protein, while the neurons were identified on the basis of their expression of the MAP2 protein. 

**Figure 6:**
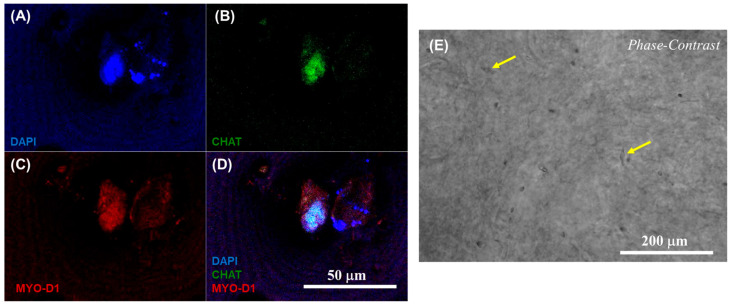
Immunostaining results confirmed via confocal microscopic imaging of 3D neurocardiac cultures (**A**–**D**), showing the AC16 cardiomyocytes (MYO D1 + Alexa Fluor 647, red) (**C**) and the differentiated SH-SY5Y neurons (CHAT + Alexa Fluor 488, green) (**B**). All cell nuclei were stained using DAPI (blue) (**A**). The merged result of both cell types is depicted in (**D**). (**E**) Phase contrast image of the neurocardiac cultures where the neuronal cell types can be seen networking with the non-neuronal cell types across the scaffold, indicated by the yellow arrows in a few places for easy identification.

Furthermore, the respective phenotypes of the neuronal and cardiac cells involved in the heterocellular cell coupling were confirmed via immunostaining using the expression of CHAT as a biomarker for differentiated neurons and MYO D1 for the cardiomyocytes. The results from confocal microscopic imaging of the immunostained 3D neurocardiac cultures are shown in Figure 6A–D, showing the AC16 cardiomyocytes (red) in Figure 6C and the differentiated SH-SY5Y neurons (green) in Figure 6B. All the cell nuclei were stained using DAPI (blue), as depicted in Figure 6A, and the merged result showing both cell types is depicted in Figure 6D. Cardiomyocytes were probed by using their expression of the MyoD1 protein, while the neurons were identified on the basis of their expression of the CHAT protein. In addition, Figure 6E shows an overview of the neurocardiac cultures, where the neuronal cell types were seen to interact with non-neuronal cell types across the scaffold. These results also confirmed that the neurocardiac coupling occurred homogeneously throughout the 3D scaffolds. 

Supplementary Figure S1 presents the immunostaining results from the 2D cultures, showing the SH-SY5Y neurons with the neurite projections identified by MAP2, and the clustering of neurons confirming their differentiation. Supplementary Figure S1C shows the cardiomyocytes cultured in the 2D controls, and identified by their expression of MyoD1. These results are presented for comparing the neuronal and cardiac cells’ morphology in the 2D culture when they were not co-cultured to confirm their identities. 

## 4. Discussion

Three-dimensional bioprinting offers several advantages over conventional tissue engineering through its potential for printing complex multicellular structures and even hollow organs. However, there are still some limitations, such as the non-uniform seeding of cells into the constructs, the placement of different types of cells in defined positions, and not being able to reconstruct complex 3D organs.

In this work, both cardiac and neuronal cell types were strategically incorporated within their respective bioinks for a semi-3D bioprinting technique. Since extrusion-based 3D bioprinting has been demonstrated to have no negative effects on cells’ survival, it has been widely used with cardiac cells [8]. Additionally, the alginate–gelatin mixture facilitated the printing process, yielding excellent support for the cardiac cells’ survival by reducing the shear stresses [28]. On the other hand, the specialized bioink for neuronal cells was comparable with the extracellular matrix present in cerebral tissue [29]. Nonetheless, given the low viscosity and the difficulty of extruding core components, microfluidic bioprinters are the most suitable option for the fabrication of neural constructs [29]. The essential benefit of this semi-3D bioprinting approach is that it eliminates the need for two different bioprinting approaches and specialized equipment for biofabricating a single structure with two types of biomaterials with differing viscosities.

Despite the low viscosity and poor mechanical handling properties of the neuronal bioink, once incorporated and crosslinked in the neurocardiac scaffold, the construct offered ease of handling and tissue culture, making it easier to manipulate during in vitro studies. Swelling and degradation analyses showed the structural integrity throughout the duration of 7 days for the composite neurocardiac scaffold. 

In order to achieve complex geometries through 3D bioprinting, it is essential for the bioink to maintain its structure and stability after printing and crosslinking. To confirm this behavior, we investigated and compared the increase in the storage moduli for both the cardiac and neuronal bioinks after crosslinking, as reported in a previously published study [30]. However, the extrusion-based cardiac bioink demonstrated a higher storage modulus, indicating more elastic-like behavior (G′) [30] when compared with the neuronal bioink [30]. The observed increase in the storage modulus in the cardiac bioink can be attributed to the sustained swelling and degradation pattern of the composite neurocardiac scaffold in comparison with the neuronal bioink alone. 

FTIR spectroscopy gathers graphical data by emitting a range of wavelengths, which specific bonds within the material absorb [23,24,25,26,27]. For instance, a wave with a wavenumber (which is the reciprocal of the wavelength) of 1050 cm^−1^ will be absorbed by the primary alcohol functional group (-OH). This is reflected in the percentage of transmittance, as the sensor at the other end of the FTIR machine records only a fraction of the initial wave sent out. The crosslinking of the hydrogels corresponding to the larger peaks was confirmed via FTIR analysis. This meant that the crosslinked bonds absorbed a greater fraction of the waves sent out compared with the non-crosslinked structures. It has been conjectured that crosslinking lowers the free energy of the functional groups, making the bonds vibrate more visibly for being recorded by FTIR spectroscopy. Moreover, the functional groups verified by FTIR spectroscopy support the idea that relatively strong intermolecular forces, such as hydrogen bonding and electrostatic attraction, arise when the two bioinks are layered upon each other. 

The cellular interactions between differentiated neurons and cardiomyocytes were emphasized by the immunostaining results. A successful heterocoupling phenomenon was demonstrated between these essential cell types, as can be found normally in physiological cardiac tissue innervated by the branches of the autonomic nervous system [31]. In previous studies, the heterocellular interactions between human induced pluripotent stem cell (hiPSC)-derived sympathetic neurons and hiPSC cardiomyocytes has been demonstrated in a 2D co-culture set-up [7]. This prior study, in comparison with our results, suggested that the adoption of a semi-3D bioprinting strategy would enhance the heterocellular coupling significantly by allowing their systematic positioning, with the cardiomyocytes placed at the bottom and the neurons at the top. Nonetheless, it is critical to further enhance this investigation and validate it with a neurocardiac cell model consisting of actual cardiac autonomic neurons along with iPSC-derived contractile cardiomyocytes to generate a functional CANS model in the future. 

Reportedly, this unique 3D bioprinting approach has not been implemented before with cardiac and neuronal cells. Owing to the lack of availability of such models, very few physiological platforms exist for understanding the mechanisms underlying the sympathetic interactions of neurons with cardiovascular units [7]. The semi-3D bioprinting approach presented in this study offers an immense potential for co-culturing cardiomyocytes and neurons by combining several cellular biomaterials into a single engineered structure. This will pave the way for the study of how neuronal regulation or dysregulation can contribute to various cardiovascular diseases, and will enable further research into how the autonomic nervous system controls heart function. This study provides a tissue on a chip platform to discover and create novel treatment approaches using 3D neurocardiac models.

## 5. Conclusions

To better understand the connections between the heart and brain tissues, a semi-3D bioprinted neurocardiac tissue model was created in this study, using cardiomyocytes combined in an alginate–gelatin hydrogel and neurons mixed in an alginate–genipin–fibrin hydrogel. The 3D scaffolds maintained the cells’ survival, function, and phenotypes while displaying structural and mechanical integrity. Additionally, the structure successfully demonstrated heterocellular interactions between the neurons and cardiomyocytes. In a variety of applications, including drug screening and tissue engineering, this semi-3D bioprinted tissue has the potential to be a useful model for examining cells’ behavior and function.

## Figures and Tables

**Table 1:** Optimized printing parameters.

Parameter	Specification
Nozzle diameter	Tapered 22G
Printing speed	1 mm/s
Pressure	70 kPa
Temperature	32 °C
Infill	25%

## Data Availability

All data analyzed or generated during the study are presented in the manuscript and in the online supplementary section.

## Supplementary Materials

The following supporting information can be downloaded at https://www.mdpi.com/article/10.3390/bioengineering10070834/s1. Figure S1: Immunostaining results from 2D cultures showing SH-SY5Y neurons (A) with neurite projections, and (B,C) showing the clustering of neurons, confirming their differentiation. (D) and (E) Cardiomyocytes cultured in the 2D controls.
