# Supplementary material for: A Semi-Three-Dimensional Bioprinted Neurocardiac System for Tissue Engineering of a Cardiac Autonomic Nervous System Model
Source: Bioengineering (Basel). 2023 Jul 14;10(7):834. doi: 10.3390/bioengineering10070834 (PMC10376081; doi:10.3390/bioengineering10070834)
Supplement: Supplementary file 1 [file bioengineering-10-00834-s001.zip › bioengineering-2475280-supplementary.pdf]

Supplementary Information

A Semi-Three-Dimensional Bioprinted Neurocardiac System for Tissue Engineering of a Cardiac Autonomic Nervous System Model

Ivana Hernandez[1,2], Salma P. Ramirez[1,2], Wendy V. Salazar[1], Sarahi Mendivil[1], Andrea Guevara[1], Akshay Patel[1,3], Zayra N. Dorado[1,2], Carla D. Loyola[1,2], and Binata Joddar[1,2,4]

[1]Inspired Materials and Stem-Cell Based Tissue Engineering Laboratory (IMSTEL), The University of Texas at El Paso, El Paso, TX, 79968, USA

[2]Department of Metallurgical, Materials, and Biomedical Engineering, M201 Engineering, The University of Texas at El Paso, 500 W. University Avenue, El Paso, TX, 79968, USA

[3]Department of Chemical Engineering, University of California, Santa Barbara, CA, 93106, USA

[4]Border Biomedical Research Center, The University of Texas at El Paso, 500 W. University Avenue, El Paso, TX, 79968, USA

Supplementary Materials and Methods

S2.1 Materials

The complete growth medium for the neurocardiac structure consisted of the following components: DMEM (Gibco 11966-025, Billings, MT), L-glutamine (Cat. No. G7513, SIGMA, St. Louis, MO), fetal bovine serum (FBS) (USDA-approved source, heat-inactivated, Cat. No. 35-011-CV, Corning, Corning, NY), penicillin–streptomycin solution (100X) (Cat. No. SV30010, HyClone, Logan, UT), glucose (Cat. No. 41095-5000, Acros Organics, Geel, BE), Nutrient Mixture F-12 Ham (Cat. No. N4888, SIGMA, St. Louis, MO), Eagle's minimum essential medium (EMEM) (Cat. No. M2279, SIGMA, St. Louis, MO), non-essential amino acids (NEAA) (Cat. No. 13-114E, Lonza, Basel, CH); Neurobasal® medium (Cat. No.21103-049, Gibco, Billings, MT); B27® Supplement Electro (50X) (Cat. No A14097-01, ThermoFisher, Scientific Waltham, MA), GlutaMAX Supplement (100X) (Cat. No. 35050-061, Gibco, Billings, MT), and trans-retinoic acid (R2625, SIGMA, St. Louis, MO).

For detaching all cells and subculturing them, the following items were used: 0.25% trypsin-EDTA (Cat. No. 25200-056, Gibco, Billings, MT), a centrifuge (ThermoFisher Scientific, Waltham, MA), T75 culture flasks (ThermoFisher Scientific, Waltham, MA), an incubator (ThermoFisher Scientific, Waltham, MA), micropipettes (ThermoFisher Scientific, Waltham, MA) (1–10 μL, 10–100 μL, 100–1000 μL), and a Countess III automated cell counter (ThermoFisher Scientific, Waltham, MA).

For confocal microscopic imaging, the samples required fixing, staining, and labeling with fluorescent dyes or antibodies to highlight the specific structures or molecules of interest. For fixing all cell–gel samples, 4% paraformaldehyde in PBS (PFA) (Cat. No. J19943-K2, ThermoScientific, Waltham, MA) was used. For cell labeling and confocal microscopic

imaging, PKH67 Green [8, 16] and PKH26 Red [8] Fluorescent Cell Linker Mini Kits were used in accordance with the manufacturer's instructions (Cat. Nos. MINI67-1KT and PKH26GL-1KT, Sigma-Aldrich St. Louis, MO). For immunofluorescence and imaging, the primary antibodies used for immunostaining included myogenic determination factor 1 (MyoD1) used at a dilution of 1:200 (ab16148, Abcam, Waltham, MA) targeted towards the AC16 cardiomyocytes, microtubule-associated protein 2 (MAP-2) added at a 1:100 dilution (131500, ThermoFisher, Waltham, MA), and recombinant Alexa Fluor 488® anti-choline acetyltransferase (CHAT) added at a 1: 200 dilution (ab192465, Abcam, Waltham, MA) for the differentiated SH-5YSY cells. The secondary antibodies used included the donkey anti-mouse IgG H&L (Alexa Fluor® 647, a bright, far red fluorescent dye) (1:800; ab150107, Abcam, Waltham, MA) to bind to MyoD1. The other secondary antibody included goat anti-mouse IgG H&L (Alexa Fluor® 488, a bright green fluorescent dye), which had been pre-adsorbed (1:200; ab150117, Abcam, Waltham, MA) to bind to MAP-2. Stained samples were mounted on microscopic glass slides (ThermoFisher, Waltham, MA) using DAPI Fluoromount-G® (Southern Biotech, Birmingham, AL) and dried prior to imaging. To visualize the three-dimensional structure of the samples and cells within them using confocal microscopy, a Z-stack of images acquired from optical sections at different focal planes was acquired. These image slices were then reconstructed into a 3D image to generate a maximum-intensity image using the software of the confocal fluorescent microscope (ZEISS LSM, Germany).

S2.5 Characterization of the Materials

S2.5.1. Swelling Analysis

The hydration behavior of acellular bioprinted scaffolds were assessed for a 1-week time period. The printed acellular scaffolds were air-dried, and the dry weights of each sample were recorded. Each sample was then immersed in 1 mL of 1X PBS at pH 7.4, without calcium and magnesium ions. The weights of the swollen gels were monitored and recorded every 24 h. A swelling ratio was determined for each sample at each time point using previously reported metrics [8]. Briefly, the data collected were used to estimate the swelling ratio by calculating the difference between the mass in the swollen state and the mass in the dried state (original weight), and normalizing this to the initial dry weight of each sample recorded.

S2.5.2 Attenuated Total Reflectance Fourier Transform Infrared Spectroscopy (ATR-FTIR)

ATR-FTIR was used to study the chemical composition, molecular structure, and functional groups present in the non-crosslinked as well as in the crosslinked samples. Acellular scaffolds were used for the ATR-FTIR absorbance studies, and measurements were carried out with the cardiac and neuronal scaffolds using a Nicolet FTIR spectrometer (ThermoFisher Scientific, Waltham, MA) equipped with a diamond ATR crystal. The spectrum of the samples was recorded from 400 to 4000 cm^{-1}, and 32 scans per sample were averaged to reduce the spectral noise.

S2.5.3 Scanning Electron Microscopy (SEM)

SEM micrographs were acquired from the acellular scaffolds to demonstrate the sequence of the semi-3D printing process adopted in this study. The cardiac scaffold was studied using SEM, followed by imaging of the neurocardiac scaffold. Visible differences were identified by analyzing the en face electron micrographs of the lyophilized acellular samples.

The 3D bioprinted scaffolds were deposited on plastic microscope slides (non-conductive) and freeze-dried or lyophilized before characterization. For freeze-drying, the samples were placed in a petri dish in a -80°C freezer overnight and lyophilized (FreeZone Triad, LABCONCO, Kansas City, MO) for 12 hours to preserve their 3D morphology. Prior to SEM imaging, the lyophilized samples were sputter-coated with gold/palladium (2–3 min) in a sputter coater (Gatan Model 682 Precision etching coating system, Pleasanton, CA) and visualized by SEM (S-3500, Hitachi, Japan) at a voltage of 15 kV in a low-vacuum setting (VP, –90 Pa) at varying magnifications. Three different sample areas were targeted to obtain the morphologies of the semi-3D bioprinted scaffolds. At least n = 5 images per sample were assessed in total.

S2.6 Cell Culture and Passaging

The AC16 human cardiomyocyte cell line (Cat. No. SCC109, EMD Millipore, Burlington, MA), derived from adult human ventricular heart tissue, was used in this study and cultured according to the vendor's recommendations. To culture these cells, normoglycemic (5 mM glucose) DMEM, a complete growth medium containing 2 mM L-glutamine, 10% FBS, and a 1X penicillin–streptomycin solution, was prepared and used.

The neuronal cells used in this study were sourced from the SH-SY5Y neuroblasts derived from human neural tissues (SH-SY5Y neuroblastoma cell line, Cat. No. CRL-2266, ATCC, city, state) [20]. The culture medium for these cells contained Ham's F12:EMEM at a1:1 ratio (EBSS), 2 mM glutamine, 1% non-essential amino acids (NEAA), 15% FBS, and 1% penicillin/streptomycin. For making the media, 250 mL of F12 was prepared using 250 mL of EMEM (EBSS), 5 mL of NEAA, 75.75 mL of FBS, 5.80 mL of glutamine (0.2M), and 5.68 mL of penicillin/streptomycin. Cells were grown and passaged for experimental stabilization as per the vendor's recommendations and seeded in a T75 flask at a seeding density of 1000–10,000 cells/cm^2. Once the desired cell density had been achieved and confirmed via cell counting, neuronal differentiation was performed adopting guidance from published studies in the literature [21]. The growth medium was replaced with a neurobasal medium containing a B27 supplement and GlutaMAX. In addition, 10 μM of all-trans-retinoic acid (ATRA) were added to the T75 culture flask to induce differentiation and the neuronal phenotypes. Cells were incubated for 3–5 days, refreshing the neurobasal medium every 48 hours (about 2 days). Morphological changes across the entire differentiation process (approximately 7 days) were monitored using an EVOS compound microscope (ThermoFisher Scientific, Waltham, MA).

The thawing process was performed as follows. The vial of AC16 cells was removed from the freezer and incubated in a 37 °C water bath until the cells had completely thawed. The outside of the vial was disinfected with 70% ethanol and transferred to the laminar flow hood. The cells were carefully transferred to a sterile 15 mL conical tube using a 2 mL pipette. Using a 10 mL pipette, 9 mL of the AC16 growth culture medium was slowly added dropwise to the 15 mL conical tube. The cell suspension was mixed slowly and centrifuged at 300xg for 3 minutes. After centrifugation, the supernatant was discarded, and the cells were resuspended in 10–15 mL of the AC16 culture medium. Then the cell mixture was transferred to a T75 culture flask, and the cells were incubated at 37 °C in a humidified incubator with 5% CO_2. The medium was exchanged the next day with 10–15 mL of fresh AC16 culture medium. Every 2–3 days after that, the medium was exchanged with fresh medium, and the cells were checked daily under a microscope for confluency. Cells were grown and passed for experimental stabilization as per vendor's recommendations. When the cells reached 80–95% confluency, they were detached from the T75 tissue culture flask using trypsin-EDTA. For passaging the cells, the growth medium was removed from the T75 tissue culture flask, and 5 mL of a trypsin-EDTA solution was added to the flask with the cells and was incubated at 37 °C for 3 minutes. Then the same volume of the AC16 culture medium was added to the T75 flask to inactivate the trypsin-EDTA plate, and the cells were detached, followed by gently rotation of the flask to ensure a homogenous cell suspension. The detached cell suspension was transferred to a 15 mL conical tube and centrifuged at 300xg for 5 minutes to form a cell pellet. The supernatant was discarded, and 2 mL of the AC16 culture medium was added to the conical tube. Using a pipette, the cells were resuspended thoroughly, and the cells were automatically counted with Countess III to estimate the cell density for further experiments.

For thawing and passaging of the neuronal cells, a similar method to that reported for the cardiac cells was adopted. For further passaging and differentiation, the growth medium in the T75 culture flask was discarded, and the adherent cells were rinsed with pre-warmed and sterile 1x PBS. Then 5 mL of trypsin was added to the adherent cells and incubated for 2 minutes or until the cells visibly detached from the culture flask. Trypsin was neutralized by adding 5 mL of the EMEM/F12 medium containing 15% FBS. The cell suspension was transferred to a 15 mL conical tube and centrifuged at 1,500 rpm for 5 minutes at room temperature to pellet the cells. The supernatant was discarded, and the pellet was suspended in 2 mL of the EMEM/F12 medium containing 15% FBS. The cell suspension was mixed by gently pipetting it up and down.

<u>Supplementary Immunostaining Results</u>

S3. Immunostaining

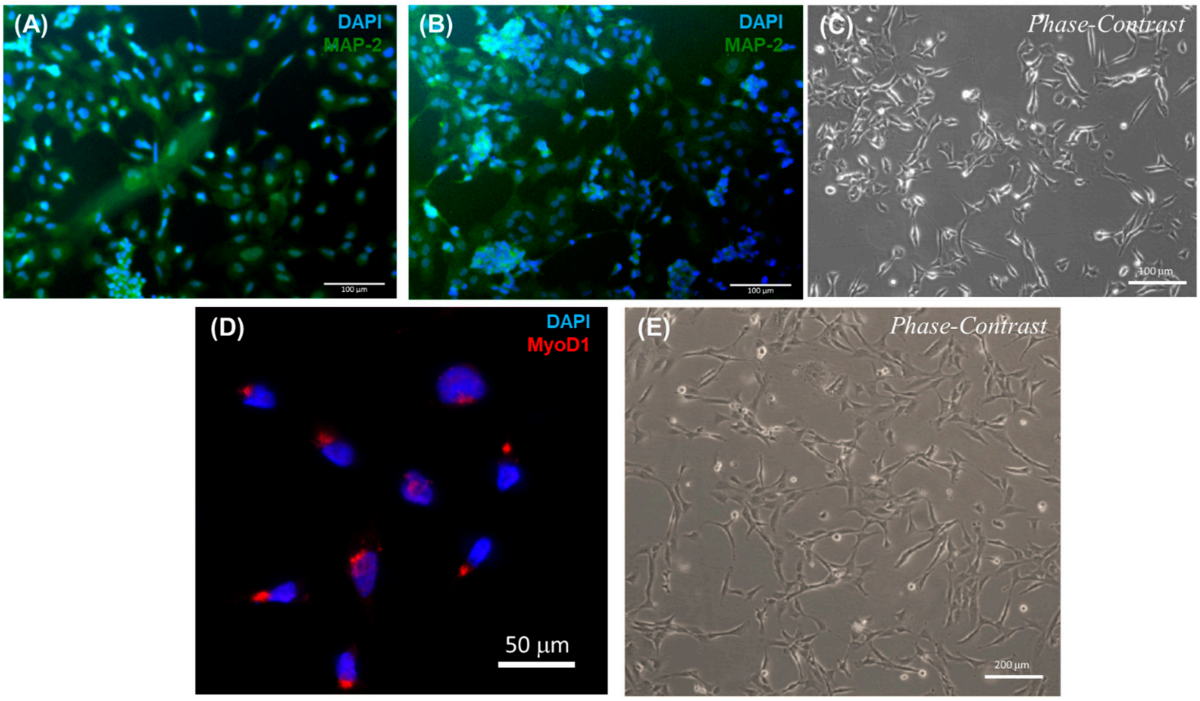

Supplementary Figure S1. Immunostaining results from 2D cultures showing the SH-SY5Y neurons (A) with neurite projections, and (B), (C) the clustering of neurons, confirming their differentiation. (D,E) Cardiomyocytes cultured in the 2D controls.
